# Comparison of Frontline FOLFIRINOX with Fluorouracil-Based and Gemcitabine-Based Chemotherapies in Metastatic Ampullary Adenocarcinoma: A Multicenter Study by the Turkish Oncology Group (TOG)

**DOI:** 10.3390/jcm14165868

**Published:** 2025-08-20

**Authors:** Ali Kalem, Tulay Kus, Savas Gokcek, Ilkay Tugba Unek, Taha Koray Sahin, Omer Dizdar, Muhammed Fatih Sagıroglu, Hatice Bolek, Yuksel Urun, Sendag Yaslıkaya, Ertugrul Bayram, Nadiye Sever, Ibrahim Vedat Bayoğlu, Omer Acar, Atike Pınar Erdogan, Seray Saray, Berkan Karabuga, Ulku Arslan Yalcıntas, Safa Can Efil, Mehmet Ali Nahit Sendur, Talat Aykut, Murat Araz, Tugce Kubra Gunes, Melike Ozcelik, Mustafa Seyyar, Gokmen Aktas, Suayib Yalcın

**Affiliations:** 1 Department of Medical Oncology, School of Medicine, Gaziantep University, Gaziantep 27310, Turkey; drtulaykus83@hotmail.com; 2 Department of Medical Oncology, School of Medicine, Dokuz Eylul University, Izmir 35220, Turkey; savagkek@yahoo.com.tr (S.G.); ilkaytugbaunek@gmail.com (I.T.U.); 3 Department of Medical Oncology, School of Medicine, Hacettepe University, Ankara 06800, Turkey; takorsah@gmail.com (T.K.S.); dromerdizdar@gmail.com (O.D.); suayibyalcin@gmail.com (S.Y.); 4 Department of Medical Oncology, Gaziantep City Hospital, Gaziantep 27100, Turkey; dr.mfsagiroglu@gmail.com (M.F.S.); mustafaseyyar27@hotmail.com (M.S.); 5 Department of Medical Oncology, School of Medicine, Ankara University, Ankara 06620, Turkey; hati.kocc@gmail.com (H.B.); yukselurun@gmail.com (Y.U.); 6 Department of Medical Oncology, School of Medicine, Çukurova University, Adana 01330, Turkey; drysendag@gmail.com (S.Y.); ertugrulbayram@gmail.com (E.B.); 7 Department of Medical Oncology, School of Medicine, Marmara University, İstanbul 34854, Turkey; dr.nadya@hotmail.com (N.S.); dr.vebay@gmail.com (I.V.B.); 8 Department of Medical Oncology, School of Medicine, Manisa Celal Bayar University, Manisa 45140, Turkey; dracaromer@gmail.com (O.A.); dr_pinarcan@yahoo.com (A.P.E.); 9 Department of Medical Oncology, Ataturk Education and Research Hospital, Balıkesir 10100, Turkey; serayyaras@hotmail.com; 10 Department of Medical Oncology, Dr. Abdurrahman Yurtaslan Oncology Education and Research Hospital, Ankara 06200, Turkey; drbkarabuga@gmail.com (B.K.); ulkuarslan63@gmail.com (U.A.Y.); 11 Department of Medical Oncology, School of Medicine, Ankara Yıldırım Beyazıt University, Ankara 06690, Turkey; scefil@hotmail.com (S.C.E.); masendur@yahoo.com.tr (M.A.N.S.); 12 Department of Medical Oncology, School of Medicine, Necmettin Erbakan University, Konya 42090, Turkey; talat_aykut@hotmail.com (T.A.); zaratarum@yahoo.com (M.A.); 13 Department of Medical Oncology, Ümraniye Education and Research Hospital, İstanbul 34764, Turkey; drtugcekubragunes@gmail.com (T.K.G.); drmelike.ozcelik@gmail.com (M.O.); 14 Gaziantep Medical Point Private Hospital, Gaziantep 27584, Turkey; aktas_gokmen@hotmail.com

**Keywords:** ampullary tumors, intestinal subtype, pancreaticobiliary subtype, metastatic, FOLFIRINOX, gemcitabine-based

## Abstract

**Background:** Adenocarcinoma arising from the ampulla of Vater is an extremely rare neoplasm, and there are limited data regarding frontline therapy for metastatic disease. We investigated the outcomes of first-line treatment with FOLFIRINOX by comparing it with other treatments in patients with advanced ampullary adenocarcinoma. **Methods:** We included 123 patients with advanced ampullary adenocarcinoma who were treated with frontline FOLFIRINOX (*n* = 32), fluorouracil (FU)-based chemotherapy (*n* = 20), and gemcitabine-based chemotherapy (*n* = 65) between August 2007 and January 2024 in this retrospective study. The median progression-free survival (mPFS) and overall survival (mOS) according to treatment and clinicopathological features were calculated using the Kaplan–Meier method. **Results:** The median age of the patients was 62 years (range, 36–78), and 75,6% of the patients had an ECOG performance status of 0–1. The mOS were 13 months (95% CI, 10.6–14.4), 11 months (95% CI, 10.6–14.4), and 12 months (95% CI, 10.6–14.4), respectively [*p* = 0.865]. There were no significant differences in OS among the chemotherapeutic agents according to histological subtypes. However, FOLFIRINOX and FU-based treatments appeared more effective in the intestinal subtype, while gemcitabine-based therapies were less effective. In the pancreaticobiliary subtype, FU-based therapies yielded a shorter outcome compared to FOLFIRINOX and gemcitabine-based therapies. Grade 3 or 4 hematologic toxicities were higher in patients treated with FOLFIRINOX. **Conclusions:** In advanced ampullary adenocarcinoma, despite higher toxicity, frontline FOLFIRINOX showed a trend toward an OS benefit in the intestinal subtype while providing a similar outcome in the pancreaticobiliary subtype.

## 1. Introduction

Ampullary cancers are part of the periampullary tumor group, and together with intestinal tumors, they have a better prognosis compared to those originating from the pancreaticobiliary region [1]. Although ampullary adenocarcinoma arises from the ampulla of Vater, it can develop from three different epithelial tissues: pancreatic, biliary tract, and intestinal [2]. These three histopathological subgroups are difficult to precisely distinguish, despite being treated with completely different systemic chemotherapy regimens. While the intestinal type, which generally has a better prognosis, can be differentiated from the pancreaticobiliary type, it is not possible to pathologically distinguish between pancreatic and biliary epithelial origins [3,4,5]. FOLFOX or FOLFIRINOX is among the preferred treatment options for intestinal cancers, but FOLFIRINOX is not considered superior to FOLFOX except in certain biological subtypes, and may therefore result in overtreatment and increased toxicity. Additionally, while FOLFIRINOX has been established as the gold standard for first-line treatment of metastatic pancreatic cancer in a phase III study comparing it with gemcitabine alone [6], it was found to be a detrimental treatment for biliary tract cancers in a phase II study [7]. On the other hand, the combination of cisplatin and gemcitabine is considered the standard backbone treatment for biliary tract cancers, while its effectiveness in pancreatic cancers is limited [8].

Given this uncertainty about the definitive histopathological subtype and the rarity of ampullary adenocarcinomas, the optimal chemotherapy regimen for these tumors remains undefined. Given this uncertainty about the definitive histopathological subtype in addition to the rarity of ampullary adenocarcinomas, the optimal chemotherapy regimen for these tumors remains undefined. There is a lack of phase III randomized controlled trials specifically targeting ampullary adenocarcinoma, and most treatment recommendations are based on case series or phase III studies involving pancreaticobiliary and intestinal system cancers. Ampullary adenocarcinoma is often grouped with either small bowel cancer or pancreaticobiliary cancer, and subgroup analyses specific to ampullary carcinoma are generally unavailable [9,10,11].

In this context, our study aims to compare FOLFIRINOX with other treatment modalities in patients with ampullary adenocarcinoma who are unsuitable for curative-intent treatments and to identify the most effective treatment modality, considering other clinicopathological parameters.

## 2. Patients and Methods

This multicenter, retrospective observational study included patients with ampullary adenocarcinoma who had unresectable or metastatic disease and were unsuitable for curative-intent treatments. The study was conducted across 13 centers in various regions of Türkiye between August 2007 and January 2024. Patients with an endoscopically visualized mass at the ampulla of Vater, pathologically confirmed as adenocarcinoma, and without a primary pancreatic mass were included. All patients were required to have been treated with either fluoropyrimidine- or gemcitabine-based chemotherapy for more than one month, to have radiographically visible disease at the time of treatment, and to have been regularly followed up with computed tomography (CT) or positron emission tomography (PET-CT) scans.

Baseline characteristics of the patients, treatment regimens, and dates of treatment initiation, progression, death, or last follow-up were recorded. The following parameters were noted: patient age, gender, comorbidities, alcohol use, smoking status, Eastern Cooperative Oncology Group (ECOG) performance status (0–2), metastatic sites, number of metastatic sites, and histopathological subtype (pancreaticobiliary type, intestinal type, or mixed type).

The following laboratory parameters were recorded: hemoglobin, neutrophil, lymphocyte, and platelet levels; albumin; globulin; detailed liver function tests; and tumor markers (CEA ng/mL, CA 19–9 U/mL).

The treatment modalities administered included the following:

- Modified FOLFIRINOX (mFOLFIRINOX)—a combination of oxaliplatin, irinotecan, leucovorin, and short-term infusional 5-fluorouracil (5-FU).
- Fluoropyrimidine (FU)-based treatments (including FUFA, capecitabine, XELOX, FOLFOX, or FOLFIRI).
- Gemcitabine-based chemotherapies (gemcitabine alone or in combination with FU or platinum agents, or nab-paclitaxel).

Additionally, radiotherapy was administered to some patients for palliative purposes.

The study was conducted in accordance with the principles outlined in the Declaration of Helsinki and received approval from the ethics committees of the participating centers. It was also approved as a multicenter study by the Gaziantep University Faculty of Medicine Ethics Committee (No. 2024/44).

## 3. Statistical Analysis

Descriptive statistics were presented as frequencies (*n*) and percentages (%). Continuous variables were expressed as means (±SD). Categorical variables were compared between groups using Fisher’s exact test and the chi-square test. The response to chemotherapy was evaluated based on the treating physician’s first response assessment.

Progression-free survival (PFS) was defined as the time from the start of chemotherapy for metastatic disease to the date of disease progression or the last follow-up. Overall survival (OS) was defined as the time from the start of chemotherapy for metastatic disease to the date of death or the last follow-up. The probabilities of survival were estimated using the Kaplan–Meier method, and the log-rank test was used to assess differences in PFS and OS according to different treatments and clinicopathological features.

Multivariate Cox proportional hazards models included all covariates with a *p*-value of <0.1 in the univariate analysis for both PFS and OS. A *p*-value of ≤0.05 was considered statistically significant. Statistical analyses were conducted using the SPSS software package, version 22.0 (SPSS Inc., Chicago, IL, USA).

## 4. Results

### 4.1. Clinical and Chemotherapy-Related Features of Patients

A total of 123 patients who received systemic chemotherapy for ampullary adenocarcinoma were analyzed. The mean age of the participants was 62 years, and 56.1% (69/123) were male. Most patients had an ECOG performance status of 0 or 1 (75.6%). The indications for chemotherapy were palliative treatment for initial metastatic disease in 61% of patients and recurrence following resection in 39%. The majority of patients had metastatic disease (82.9%), with common sites of metastasis including the liver, other visceral organs, bone, and peritoneum. Among the patients, 65% had the pancreaticobiliary subtype, while 23.6% had the intestinal subtype. Other clinicopathological features are shown in Table 1.

A total of 26% of patients were treated with FOLFIRINOX, and another 26% received a platinum–gemcitabine combination. Details of the FU-based and gemcitabine-based treatments are shown in Table 1. There were no significant differences in baseline characteristics among patients treated with FOLFIRINOX, FU-based treatments, and gemcitabine-based treatments, except for the stage of disease (Table 2).

Second-line chemotherapy was administered to 52.1% (64/123) of the patients. The most common second-line regimens were FU-based therapies (62.7%), followed by gemcitabine-based treatments.

### 4.2. Progression-Free Survival and Overall Survival According to Type of Chemotherapy

**Figure 1:**
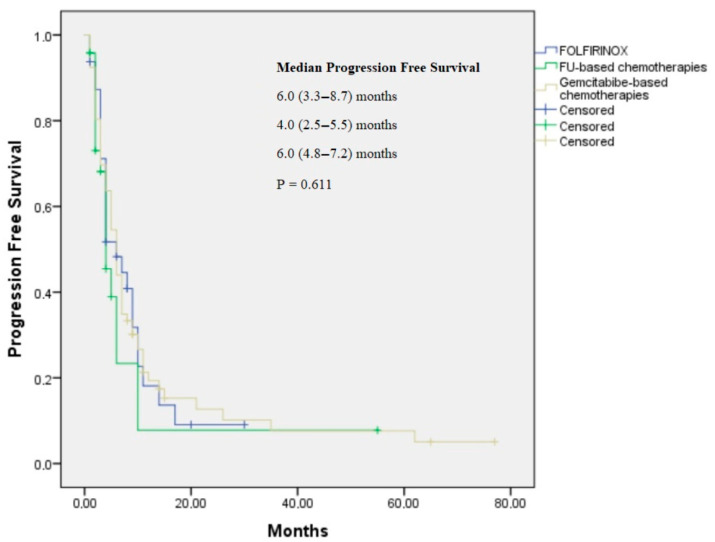
Kaplan–Meier curve for progression-free survival by histopathological subtype for FOLFIRINOX, FU-based, and gemcitabine-based treatment arms.

**Figure 2:**
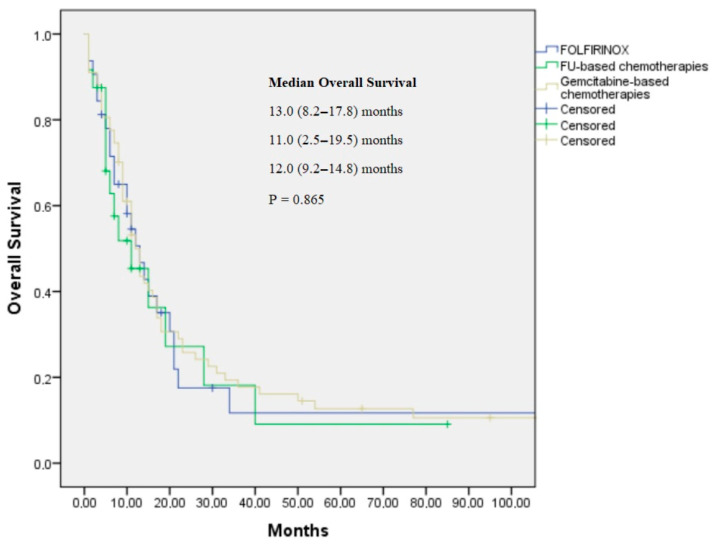
Kaplan–Meier curve for overall survival by histopathological subtype for FOLFIRINOX, FU-based, and gemcitabine-based treatment arms.

The median follow-up period was 11.0 months. A total of 32 patients were treated with FOLFIRINOX, 24 patients with fluoropyrimidine-based chemotherapy, and 67 patients with gemcitabine-based therapy. The objective response rates (ORRs) were 40.0%, 33.3%, and 29.8% for the FOLFIRINOX, FU-based, and gemcitabine-based treatment arms, respectively. FOLFIRINOX, FU-based chemotherapy, and gemcitabine-based treatment provided similar PFSs (6.0, 4.0, and 6.0 months, respectively; *p* = 0.196) and OS benefits (13.0, 11.0, and 12.0 months, respectively; *p* = 0.86) (Table 3; Figure 1 and Figure 2).

According to the histological subgroup, the mPFS was similar between groups (5.0 months; range, 2.7–6.2 months in the intestinal subgroup vs. 6.0 months; range, 3.8–6.2 months in the pancreaticobiliary subgroup; (*p* = 0.459). However, OS was significantly longer in the intestinal subgroup compared to the pancreaticobiliary subgroup (18.0 months; range, 6.9–29.1 vs. 11.0 months; range, 9.1–12.9; *p* = 0.001).

When patients were grouped by histological subtype, there was no statistically significant difference in either mPFS (*p* = 0.26) in the intestinal subgroup and (*p* = 0.10) in the pancreaticobiliary subgroup) or mOS (*p* = 0.84) in the intestinal subgroup and (*p* = 1.88) in the pancreaticobiliary subgroup) among the three treatment arms. However, survival duration was longer in the FOLFIRINOX group compared with the gemcitabine-based group in the intestinal subtype group (34.0 months; range, 10.0–58.0 vs. 13.0 months; range, 4.5–21.8, respectively) (Table 4). Among the three arms, FU-based therapies yielded worse overall survival in the pancreaticobiliary subgroup (6.0 months; range, 2.8–9.2). FU-based treatments may be an option for the intestinal subtype (where mOS was not reached), but they appear unsuitable for the pancreaticobiliary subtype. Similarly, gemcitabine-based therapies demonstrated shorter survival times for the intestinal subgroup (13.0 months; range, 4.5–21.8), while they may be preferred over FOLFIRINOX treatment with similar survival outcomes in the pancreaticobiliary subgroup (12.0 months; range, 8.9–15.1 vs. 11.0 months; range, 4.4–17.6, respectively).

Fifty-eight patients (47.2%) received second-line treatment. In second-line therapy, 27 patients received FU-based regimens, 21 were treated with gemcitabine-based therapy, and 4, 5, and 1 patients received FOLFIRINOX, taxane-nab-paclitaxel, and pembrolizumab, respectively. A total of 19 patients (15.4%) were analyzed for the presence of mismatch repair deficiency; 18 were evaluated as microsatellite-stable (MSS), while 1 patient was detected as MSI-high and received pembrolizumab as second-line treatment. Next-generation sequencing (NGS) was performed on 10 patients (8.1%); no mutations were observed in 6 patients, while EGFR fusion was detected in 1 patient, FGFR3 fusion in 1 patient, PIK3CA mutation in 1 patient, and SMAD4 and TP53 mutations in 1 patient. None of these patients received targeted therapy.

Among the clinicopathological features, alcohol use, de novo disease, and the type of metastatic site (such as liver-only or bone metastasis) were associated with worse survival, particularly in the pancreaticobiliary subtype (Table 3). Additionally, high levels of CEA (9.0 vs. 14.0 months; *p* = 0.019) and CA 19-9 (9.0 vs. 15.0 months; (*p* = 0.001) were associated with poor prognosis.

### 4.3. Toxicity

Toxicity data were obtained for 108 patients. Dose reductions due to toxicity were required in 53.8% of patients in the FOLFIRINOX treatment arm, compared to 25% in the FU-based treatment arm and 15.5% in the gemcitabine-based arm (*p* = 0.001). Treatment interruptions occurred in 30.8% of the FOLFIRINOX arm, 16.7% of the FU-based arm, and 17.2% of the gemcitabine-based arm (*p* = 0.319). Treatment discontinuation due to toxicity was observed in 11.5% of patients receiving FOLFIRINOX, 0% receiving FU-based therapy, and 6.9% receiving gemcitabine-based therapy (*p* = 0.249).

Grade 3 or higher neutropenia occurred in 23.1% of the FOLFIRINOX arm, 16.7% of the FU-based arm, and 13.8% of the gemcitabine-based arm (*p* = 0.90). Grade 3 or higher anemia was observed in 4% of the FOLFIRINOX arm, 9.1% of the FU-based arm, and 1.8% of the gemcitabine-based arm (*p* = 0.675). Grade 3 or higher thrombocytopenia rates were 12% for the FOLFIRINOX arm, 4.5% for the FU-based arm, and 5.3% for the gemcitabine-based arm (*p* = 0.608).

The rates of febrile neutropenia were similar across all arms (15.5%, 12.5%, and 10.3% for FOLFIRINOX, FU-based, and gemcitabine-based therapies, respectively; (*p* = 1.00). Grade 3 or higher liver function test deterioration was observed in 7.7% of the FOLFIRINOX arm, 0% of the FU-based arm, and 1.8% of the gemcitabine-based arm (*p* = 0.070). Grade 3 or higher diarrhea was observed in 16% of the FOLFIRINOX arm, 0% of the FU-based arm, and 5.6% of the gemcitabine-based arm (*p* = 0.124). Grade 3 or higher stomatitis was observed in 4.0% of the FOLFIRINOX arm, 4.5% of the FU-based arm, and 0% of the gemcitabine-based arm (*p* = 0.267).

## 5. Discussion

There is no consensus on the optimal management of patients with metastatic ampullary adenocarcinoma. Due to the lack of high-level evidence and prospective randomized studies, most of our knowledge regarding ampullary carcinoma is derived from case series or phase II studies with small sample sizes [1,12,13,14]. As a result, the treatment of ampullary carcinoma is often aligned with strategies for small bowel cancer or pancreaticobiliary cancers. In this study, we analyzed the efficacy of FOLFIRINOX treatment considering the histopathological subgroups. FOLFIRINOX demonstrated effectiveness in both the intestinal and pancreaticobiliary subgroups, suggesting it as a viable treatment option. Although dose reduction was more frequent in the FOLFIRINOX arm, as expected, the toxicity profile was not worse than those observed with FU-based or gemcitabine-based treatments. However, FU-based treatments were less effective in the pancreaticobiliary subgroup, while gemcitabine-based treatments were less effective in the intestinal subgroup. This highlights the importance of histological subtyping in selecting between FU-based or gemcitabine-based therapies. Our study represents one of the largest case series on this topic, providing new insights into the efficacy of FOLFIRINOX in this rare cancer type.

We can consider the data of a limited number of phase II and phase III studies. In the ABC-02 phase III study, ampullary cancers were analyzed as a type of biliary tract cancer, and the addition of platinum to gemcitabine demonstrated both PFS and OS benefits (11.7 vs. 8.1 months; HR 0.64 [95% CI, 0.52–0.80]; *p* < 0.001) [9]. However, with only 4–5.3% of cases being ampullary cancers, specific conclusions cannot be drawn. In a phase II study by conducted Kim ST et al., the effects of cisplatin-based doublet chemotherapy (with FU or gemcitabine) were analyzed in 29 patients with ampullary adenocarcinoma, obtaining an mPFS of 4.9 months (95% CI, 3.4–6.4) and an mOS of 12.5 months (95% CI, 10.6–14.4), without significant differences between chemotherapeutic agents [15]. However, this study did not consider histopathological subtypes. Our findings, with a median OS of 12.0 (9.2–14.8) months for gemcitabine-based treatments for all subtypes, are consistent with these studies. When considering histopathological subtypes, a better mOS was achieved in the intestinal subgroup with FU-based and FOLFIRINOX treatments compared to gemcitabine-based treatments, although this was not statistically significant. Additionally, gemcitabine-based treatment and FOLFIRINOX yielded better overall survival compared to FU-based treatment in the pancreaticobiliary subtype (12.0, range, 8.9–15.1; 11.0, range, 4.4–17.6; and 6.0, range, 2.8–9.2, respectively) (Table 4). This underscores the importance of histological subtyping when selecting gemcitabine-based and FU-based treatments.

In our study, the median OS was 18 months (range, 6.9–29.1) for the intestinal subtype compared to 11.0 months (range, 9.1–12.9) for the pancreaticobiliary subtype (*p* = 0.001), making histological subtype one of the most important and significant prognostic factors. Therefore, different responses to treatments are possible depending on the histological subtype. A retrospective study by Kim HS et al. on 17 patients demonstrated that XELOX treatment was more effective in the intestinal subgroup than in the pancreaticobiliary subgroup (mPFS: 13.1 vs. 6.4 months, respectively; (*p* = 0.038), aligning with our results [16]. In the present study, in the intestinal subtype, the mPFS was 4 months (1.0–7.0) for FU-based treatments, lower than for FOLFIRINOX (10.0 months; range, 1.1–18.9), but mOS was not analyzed as three out of five patients had no events. The low number of patients included in our study limited the interpretation of FU-based treatments in the intestinal subtype. FOLFIRINOX showed longer survival in this group, with an mOS of 34 months (range, 10–58) compared to gemcitabine-based treatments (13.0, range, 4.5–21.8 months), although it did not reach statistical significance, likely due to the small sample size. Conversely, FU-based treatments were administered to 15 patients in the pancreaticobiliary subtype and demonstrated significantly poorer survival compared to other treatments, with an mOS of 6.0 months (range, 2.8–9.2). Therefore, FU-based treatments do not appear suitable for the pancreaticobiliary subgroup.

A large multicenter cohort study showed that the median OS of patients with locally advanced ampullary carcinoma was 19.8 months, and the median PFS for frontline chemotherapy was 7.6 months, consistent with our results. No statistically significant differences in OS and PFS were observed between groups treated with platinum–gemcitabine and XELOX (20.4 months vs. 16.0 months, *p* = 0.341; 8.4 months vs. 5.1 months, *p* = 0.279, respectively) [17]. However, this study did not evaluate efficacy based on histopathological subtype. The National Comprehensive Cancer Network (NCCN) guidelines recommend FOLFIRINOX or gemcitabine-based chemotherapy for the pancreaticobiliary type and FOLFOX, FOLFIRINOX, or FOLFIRI for the intestinal type [18]. However, there is no specific data or study cited by the NCCN to support these recommendations based on histological subtype. Our study provides evidence for the importance of considering histological subtypes in treatment selection and offers data that could support the NCCN guidelines.

The main limitations of this study include its retrospective design, the limited number of cases, and the heterogeneity of the FU-based and gemcitabine-based treatment arms compared to the FOLFIRINOX arm. However, given the rarity of this cancer type, the data obtained in our study can provide valuable guidance to clinicians in treatment selection and offer insights that could help inform future guideline recommendations. For the first time, even retrospectively, the efficacy of FOLFIRINOX has been analyzed, demonstrating its potential as an important treatment option for both intestinal and pancreaticobiliary metastatic ampullary adenocarcinomas.

## Figures and Tables

**Table 1:** Clinicopathologic characteristics of the patients and treatments.

Clinicopathological Features	N (%)
Age	
Median (range)	62 (37–83)
<65	79 (64.3)
≥65	44 (35.7)
Gender	
Female	54 (43.9)
Male	69 (56.1)
ECOG PS	
0–1	93 (75.6)
2	13 (10.6)
Unknown	17 (13.8)
Comorbidity	
Present	41 (33.3)
Absent	67 (54.5)
Smoker	41 (33.3)
Non-smoker	64 (52.0)
Alcohol use	7 (5.7)
None	98 (79.7)
De novo metastatic	75 (61.0)
Recurrence	48 (39.0)
Stage	
Unresectable	21 (17.1)
Metastatic	102 (82.9)
Histologic subtype	
Intestinal	29 (23.6)
Pancreaticobiliary	80 (65.0)
Unknown	14 (14.4)
Site of metastasis	
Local recurrence/local LAP	21 (17.1)
Only liver	53 (43.1)
Bone	7 (5.7)
(with or without other site)	
Other visceral metastasis	24 (19.5)
(with or without liver)	
Peritoneal metastasis	16 (13.0)
(with or without other site)	
Number of metastatic sites	
0	21 (17.1)
1	84 (68.3)
≥2	18 (14.6)
Obstructive jaundice, *n* (%)	
Present	50 (40.7)
Absent	73 (59.3)
CEA ng/mL	
0–5	78 (63.4)
>5	41 (33.3)
Unknown	4 (3.3)
CA 19–9 U/mL	
<150	74 (60.2)
≥150 (if bilirubin is high: 300)	45 (36.6)
Unknown	4 (3.3)

**Table 2:** Clinicopathological features among the three treatments arms.

Clinicopathological Features	FOLFIRINOX	FU-Based	Gemcitabine-Based	*p*-Value
Age				
<65	23 (71.9)	14 (58.3)	42 (62.7)	0.536
≥65	9 (28.1)	10 (41.7)	25 (37.3)	
Gender, *n* (%)				
Female	19 (31.2)	10 (41.7)	34 (50.7)	0.182
Male	22 (68.8)	14 (58.3)	33 (49.3)	
ECOG PS				
0–1	24 (92.3)	20 (83.3)	49 (87.5)	0.62
2	2 (7.7)	4 (16.7)	7 (12.5)	
Comorbidity, *n* (%)				
Present	13 (50.0)	12 (50.0)	28 (48.3)	1
Absent	13 (50.0)	12 (50.0)	30 (51.7)	
Smoker	10 (38.5)	9 (39.1)	22 (39.3)	1
Non-smoker	16 (61.5)	14 (60.9)	34 (60.7)	
Alcohol use	0 (0)	1 (4.3)	6 (10.7)	0.192
None	26 (100)	22 (95.7)	50 (89.3)	
Stage, *n* (%)				
De novo metastatic	21 (34.4)	12 (50.0)	42 (62.7)	0.45
Recurrence	11 (65.6)	12 (50.0)	25 (37.3)	
Stage				
Locally advanced	10 (31.2)	5 (20.8)	6 (9.0)	0.019
Metastatic	20 (68.8)	19 (79.2)	61 (91.0)	
Histologic subtype, *n* (%)				
Intestinal	9 (28.1)	5 (25.0)	15 (26.3)	1
Pancreaticobiliary	23 (71.9)	15 (75.0)	42 (73.7)	
Site of metastasis, *n* (%) Local recurrence/local LAP Only liver Bone (with or without other site) Other visceral metastasis (with or without liver) Peritoneal metastasis (with or without other site)	10 (31.2) 13 (40.6) 2 (6.2) 7 (21.9) 0	5 (20.8) 10 (41.7) 1 (4.2) 4 (16.7) 4 (16.7)	6 (9.2) 30 (46.2) 4 (6.2) 13 (20.0) 12 (18.5)	0.063
Number of metastatic sites				
0	10 (31.2)	5 (20.8)	6 (9.0)	0.082
1	18 (56.2)	15 (62.5)	51 (76.1)	
≥2	4 (12.5)	4 (16.7)	10 (14.9)	
Obstructive jaundice, *n* (%)				
Present	14 (43.8)	6 (25.0)	30 (44.8)	0.22
Absent	18 (56.2)	18 (75.0)	37 (55.2)	
CA 19–9 U/mL				
<150	19 (61.3)	16 (69.6)	39 (60.0)	0.71
≥150 (if bilirubin is high: 300)	12 (38.7)	7 (30.4)	26 (40.0)	
CEA ng/mL				
0–5	19 (59.4)	19 (82.6)	40 (62.5)	0.165
>5	13 (40.6)	4 (17.4)	24 (37.5)	

**Table 3:** The median progression-free survival and overall survival according to clinicopathological features and systemic treatments.

Clinicopathological Features	Progression-Free Survival (Median, mo) (95%CI)	*p*-Value	Overall Survival (Median, mo) (95%CI)	*p*-Value
Age		0.22		0.262
<65	6.0 (4.5–7.5)		13.0 (10.2–15.8)	
≥65	4.0 (2.5–5.5)		11.0 (5.9–16.1)	
Gender		0.97		0.98
Female	6.0 (4.2–7.8)		11 (8.9–13.1)	
Male	6.0 (4.5–7.5)		13.0 (10.5–15.5)	
ECOG PS		0.387		0.88
0–1	5.0 (3.8–6.2)		11.0 (9.2–12.8)	
2	4.0 (1.2–6.8)		29.0 (0.0–56.8)	
Comorbidity		0.46		0.84
Present	5.0 (3.8–6.3)		11.0 (7.9–14.1)	
Absent	6.0 (3.9–8.1)		12.0 (8.9–15.1)	
Smoker	5.0 (3.6–6.4)	0.84	9.0 (6.6–11.4)	0.29
Non-smoker	6.0 (4.4–7.6)		12.0 (9.9–14.1)	
Alcohol use	6.0 (4.8–7.2)	0.038	7.0 (4.4–9.6)	0.034
None	4.0 (0–9.1)		12.0 (9.9–14.1)	
De novo disease	6.0 (4.6–7.4)	0.62	11.0 (9.1–12.9)	0.017
Recurrence	6.0 (4.3–7.7)		15.0 (4.6–25.4)	
Stage		0.142		0.163
Locally advanced	6.0 (1.9–10.1)		13.0 (3.3–22.7)	
Metastatic	5.0 (3.8–6.2)		12.0 (9.9–14.1)	
Histologic subtype		0.459		0.001
Intestinal	5.0 (2.7–7.3)		18.0 (6.9–29.1)	
Pancreaticobiliary	6.0 (4.3–7.7)		11.0 (9.1–12.9)	
Site of metastasis		0.141		0.046
Local recurrence/local LAP	6.0 (1.9–10.1)		13.0 (3.2–22.7)	
Only liver	6.0 (4.3–7.7)		9.0 (6.3–11.6)	
Bone	4.0 (3.4–4.66)		9.0 (1.3–16.7)	
(with or without other site)				
Other visceral metastasis	6.0 (4.2–7.8)		17.0 (12.6–21.4)	
(with or without liver)				
Peritoneal metastasis	5.0 (3.6–6.4)		12.0 (1.0–23.0)	
(with or without other site)				
Number of metastatic sites		0.001		0.219
0	6.0 (1.9–10.1)		13.0 (3.3–22.7)	
1	6.0 (4.6–7.4)		13.0 (9.9–16.0)	
≥2	4.0 (3.3–4.7)		9.0 (3.3–14.7)	
Obstructive jaundice		0.69		0.25
Present	6.0 (4.4–7.6)		11.0 (7.4–14.6)	
Absent	6.0 (4.3–7.7)		13.0 (9.5–16.4)	
CA 19–9 U/mL		0.008		0.001
<150	6.0 (4.6–7.4)		15.0 (11.9–18.1)	
≥150 (if bilirubin is high: 300)	4.0 (3.1–4.9)		9.0 (7.8–10.2)	
CEA ng/mL		0.196		0.019
0–5	6.0 (4.3–7.7)		14.0 (9.9–18.1)	
>5	4.0 (3.3–4.7)		9.0 (6.4–11.5)	
Treatments		0.611		0.865
FOLFIRINOX	6.0 (3.3–8.7)		13.0 (8.2–17.8)	
FU-based	4.0 (2.5–5.5)		11.0 (2.5–19.5)	
Gemcitabine-based	6.0 (4.8–7.2)		12.0 (9.2–14.8)	

**Table 4:** The median progression-free survival and overall survival according to histological subtypes among the three chemotherapy arms.

Histologic Subtype	Treatments, (*n*)	Progression-Free Survival (Median, mo) (95% CI)	*p*-Value	Overall Survival (Median, mo) (95% CI)	*p*-Value
Intestinal type	FOLFIRINOX (9)	10.0 (1.1–18.9)	0.47	34.0 (10.0–58.0)	0.84
	FU-based (5)	4.0 (1.0–7.0)		NR (NR–NR)	
	Gemcitabine-based (23)	5.0 (2.5–7.5)		13.0 (4.5–21.8)	
Pancreaticobiliary type	FOLFIRINOX (23)	4.0 (2.0–6.0)	0.071	11.0 (4.4–17.6)	1.88
	FU-based (15)	4.0 (2.4–5.6)		6.0 (2.8–9.2)	
	Gemcitabine-based (42)	7.0 (5.5–8.5)		12.0 (8.9–15.1)	

## Data Availability

The original contributions presented in this study are included in the article. Further inquiries can be directed to the corresponding author. The datasets analyzed are available from the corresponding author on reasonable request.
